# Forensic maceration – A comparative analysis of literature and practical application

**DOI:** 10.1007/s00414-025-03684-y

**Published:** 2026-01-02

**Authors:** Melissa Kirbach, Caroline Kohlt, Wilma Ludvigsson Möller, Markus Alexander Rothschild, Anja Petaros

**Affiliations:** 1 Institute of Legal Medicine, Medical Faculty, University of Cologne, Melatengürtel 60/62, 50823 Cologne, Germany; 2 Forensic Science Institute, Bundeskriminalamt (BKA), Äppelallee 45, 65203 Wiesbaden, Germany; 3 National Forensic Centre, Swedish Police Authority, Brigadgatan 13, 58194 Linköping, Sweden; 4 Division of Forensic Medicine Linköping, National Board of Forensic Medicine, Artillerigatan 12, 58758 Linköping, Sweden; 5 Division of Clinical Chemistry and Pharmacology, Department of Biomedical and Clinical Sciences, Faculty of Medicine, Linköping, Sweden

**Keywords:** Maceration, Forensic pathology, Forensic anthropology, Tool marks, Material traces, Bone

## Abstract

In forensic practice, the removal of soft tissue is sometimes required to allow for a more accurate skeletal analysis. One of the preferred skeletal processing methods is maceration, the softening of tissue by soaking in water. Despite being widely mentioned in forensic literature, there is still a lack of comprehensive data on how and which maceration methods are applied in daily forensic practice, especially as maceration can be carried out by different forensic professionals and in diverse forensic settings. The aim of this study was to review the published literature on forensic use of maceration and conduct an international survey to compare the practices used with those described in the literature, with special attention on the effects on tool marks and material traces analyses on bone. The literature review, conducted on 27 articles that met the study’s inclusion criteria, showed that maceration has been a topic of research and methodological development over the years. Warm water maceration and detergent-based methods were recognized as preferred for practical use, even though no maceration method is without limitations. Survey responses from 57 laboratories and institutes from 19 different countries indicated that while most used methods align with literature recommendations, some practices considered aggressive remain in use. Additionally, it was observed that there is a significant lack of standardization, and maceration is often not included in standard operating procedures (SOPs), which can affect the consistency, efficiency and reproducibility of the methods used, which is something that should be addressed in future.

## Introduction

The examination and analysis of bones provide extensive and valuable findings across numerous scientific disciplines. Bones are analyzed in various forensic fields such as forensic medicine, forensic anthropology and archaeology, tool mark analysis and material trace examination [1].

By examining bones, the forensic significance of a finding can be assessed, the biological profile of unknown skeletal remains reconstructed, identification features recorded, and, in cases of trauma, the type of trauma, it’s mechanism and the sequence of traumatic events determined [1–5]. Bone analysis also enables differentiation between postmortem (taphonomical) damage [6] and perimortem trauma. In cases of trauma, visual analysis can often reveal the characteristic features of the tool that produced it. Class and subclass characteristics can be utilized to narrow down the type of the tool involved [7], and in some cases, based on individualizing characteristics in the marks left on the bone surface, the identification of a specific object as the instrument that caused the lesion can be achieved [8–18]. Bone lesions and contact surfaces can retain material traces (lacquer, metal abrasion etc.) from the tool used to inflict it, which can provide important information for trauma interpretation [19, 20].

Human bones can be analyzed in a variety of forensic scenarios: from fully skeletonized remains to those examined in the immediate aftermath of death. Skeletal analysis may also be performed on remains in various stages of decomposition and on those affected by different taphonomic agents, including fire, soil, plant or animal activity, or human modification such as dismemberment or chemical alteration (e.g. exposure to acids) [21].

In many of these scenarios, removal of tissue may be necessary to facilitate an optimal skeletal analysis.

Maceration is one of the most used skeletal processing techniques for this purpose, specifically aimed at the removal of soft tissue, typically through soaking [22] (some authors, however, extend the term to include mechanical and entomological methods- these are however less commonly used in forensic contexts, since the former carries a risk of damaging the bone and leaving tissue residues, while the latter requires the maintenance of insect colonies and is difficult to standardize and control). The maceration methods currently used in forensics have derived from techniques developed originally for taxidermy and anatomical preparations but differ from them in their purpose and outcome. Forensic maceration aims to preserve bone integrity and minimize alterations to bone structure and traces, while also ensuring long-term storage to facilitate future analyses and investigations. In taxidermy and anatomical preparations, the bones can be significantly whitened, defatted, and consolidated following maceration, to enhance their appearance and longevity, which is often not the case in forensic maceration. In certain cases, depending on the regulations and standards in place, forensic maceration may also need to be rapid, as skeletal parts might need to be returned to the body before it is released to the relatives for burial. Besides, once macerated, the bones in forensic cases may be handed over to other specialists, who can focus on other aspects of the remains than those initially evaluated, or be subjected to additional analysis and sampling (e.g., in cold cases). That is why it becomes crucial that maceration is conducted in a way that minimizes alteration and preserves all relevant details as thoroughly as possible.

Maceration methods are typically categorized into cold and hot water maceration, chemical methods, and enzymatic methods (used either alone or within detergents) [23, 24]. Cold-water maceration refers to soaking remains in water at room temperature, often for extended periods. In contrast, hot-water maceration refers to maceration by soaking at elevated temperatures without boiling, which accelerates the removal of soft tissues. Chemical maceration refers to the use of chemical agents, most commonly detergents, often in a heated water bath, to facilitate the removal of soft tissues. This category also includes the use of more aggressive oxidizing agents (e.g., hydrogen peroxide, bleach) or alkaline solutions (e.g., sodium hydroxide), which act faster but may damage bone integrity. Enzymatic maceration refers to the use of enzymes without additional chemical additives.

Even if maceration represents an important technique for skeletal preparation, it is still unclear how often it is performed, as there is no comprehensive data or statistics available. There is also insufficient information on the preferred maceration methods and approaches used in practice, as well as on the standards, if any, followed across forensic laboratories. This study addresses the latter issue through two approaches: a comprehensive review of the scientific literature on maceration in forensic settings, aiming at assessing and comparing the methods studied and the conclusions drawn; and a structured questionnaire for practitioners who perform maceration as part of their work, mainly in forensics. The questionnaire aims to gather information on maceration methods used in different forensic institutes/countries, whether protocols are followed, and if these methods are incorporated into standard operating procedures (SOPs), considering also tool mark and material trace analysis. Finally, the study compares practitioners’ methods with findings from the literature.

## Methods

The systematic review of the scientific literature on forensic maceration was conducted sourcing articles from databases Google Scholar and PubMed up to October 2024. Keywords used for the primary search included "maceration" or “preparation” combined with "bone," “skeletal”, "forensic", “forensic pathology” and "forensic anthropology". The inclusion criteria were studies published in international journals that employed, compared or tested enzymatic, chemical, or water-based maceration methods (maceration in the narrow sense, thus excluding entomological and mechanical preparation techniques) on human remains or non-human proxies for use in forensic settings. Articles on anatomical and museum specimen preparation or preparation of non-bone tissue (cartilage), articles on preparation/maceration of formaldehyde treated body parts/embalmed corpses, non-English publications, book chapters, and texts without full access were excluded from the review.

To identify studies that met the established inclusion criteria and the aim of the study, titles and abstracts of articles from the initial search (totaling 468 articles) were screened, and articles aligning with the aim of the study were selected. Subsequently, a cross-reference of citations from the selected articles was conducted to identify any additional relevant publication.

The survey was conducted as part of the ISF-funded project (HIEB), to gather information on various maceration methods used in different forensic institutes. Two questionnaires, available in both German and English, were developed and distributed to practitioners dealing with skeletal preparations and analysis. Participants were reached through forensic anthropology professional societies or their active members (e.g., FASE, AAFS), as well as by internet searches and internal professional contacts, and encompassed two distinct target groups:

FM: Institutes of forensic medicine, forensic anthropology, archaeology, and anatomy.

FSL: Forensic science laboratories including national institutes and those belonging to the European Network of Forensic Science Institutes (ENFSI).

Since the survey was initiated in Germany, the two target groups were defined based on the division of tasks commonly applied there, where forensic medicine practitioners perform the autopsy and conduct macerations, whereas the forensic science laboratories handle tool mark and material trace analysis. Based on their different tasks the questions were formulated to fit their area of responsibility.

Based on reliability, safety, data protection, privacy, and access control, the platform soscisurvey.de was chosen to implement the online survey. The survey was distributed by a blind copy to the chosen participating institutes via a link and a QR code. For each institute one responsible practitioner answered the questionnaire.

The survey started on March 06th, 2024 and closed July 15th, 2024. It included different sets of questions (Table 1 and Table 2) for each participant group (FM and FSL, respectively). The questions were formulated either as open-text fields (orange), as multiple-choice options with the possibility of multiple selections (pink) or as exclusion questions (yes/no questions, blue). For the open-text field questions, possible answers were provided to facilitate completion.

**Table 1.**
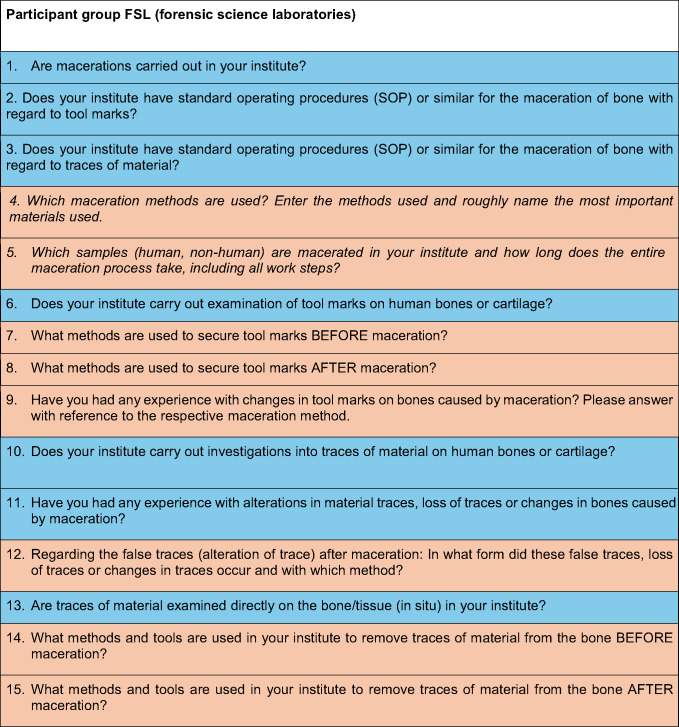
Questions addressing the FSL target group (questions that are the same for both target groups are written in italics.)

**Table 2.**
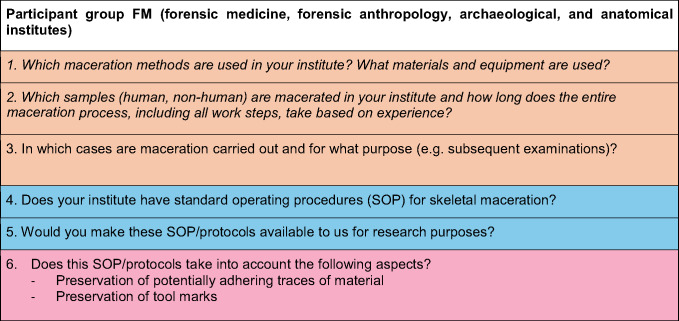
Questions addressing target group FM (Questions from both participant groups that have the same content are written in italics.)

## Results

### Literature review

The literature search resulted in a final set of 28 articles that met the defined inclusion criteria and aligned to the aims of the study. Of these, the authors were able to fully access 27 articles ([24–50]), which were reviewed in detail. A comprehensive overview of the methods evaluated, and conclusions drawn for each study are summarized in the Supplementary Table 3 (Appendix A Table 4). Post-maceration treatment, such as degreasing and bleaching, has not been included or further considered, as they were not mentioned consistently across articles.

The reviewed articles were published between 1975 and 2024. Most studies focused on presenting a new method for processing human remains (10/27) and/or comparing different maceration methods (10/27) to evaluate the most effective one, ranging from a minimum of two to a maximum of ten methods per study.

Of the 27 reviewed studies, detergent maceration was the most commonly evaluated/presented, appearing in 17 studies. Water bath maceration (without additives) was used in 14 studies, followed by maceration by sodium hypochlorite (bleach- a strong alkaline and oxidizing agent), employed in ten studies, and enzyme maceration, used in nine studies. Less commonly tested were the microwave maceration (included in four studies), hydrogen peroxide and borax maceration (each included in two studies), as well as maceration with antiformin (i.e. sodium hypochlorite solution; appearing in just one study from 1975 [25]).

The methods were tested primarily on human remains (14/27). When animal proxies were used, porcine specimens were the most commonly utilized (9/27).

Temperature settings varied both across and within studies and within maceration methods. Water bath maceration was evaluated at temperatures ranging from room temperature (“cold water maceration”, approximately 21–24 °C) to simmering (at around 90 °C) and boiling points (100 °C). For that both tap water and deionized water (implemented in one study) were used. Sodium hypochlorite (bleach) was tested in both undiluted form and in 5–10% dilutions, typically at room temperature but also at temperatures ranging from 15 °C to 70 °C. Detergent-based maceration methods used various commercial products, from household laundry detergents, dishwashing detergents and soaps to heavy duty detergents with or without the addition of sodium carbonate or meat tenderizer at temperatures ranging from room temperature up to 100 °C. The enzyme-based methods included both commercial enzyme mixtures (sometimes of undefined content) and single enzymes, most often protease and lipase. These have been evaluated at different temperatures, ranging from room temperature to 60 °C, but most commonly around 40 °C.

Among the studies that were comparing different maceration methods, three specifically examined methods to be applied in particular forensic contexts (e.g., neonatal remains [24] and burned remains [49]).

Studies evaluated the methods for their usefulness and applicability in forensic settings, evaluating factors such as efficiency, processing time, ease of use, and impact on bone quality, most often relying on predefined ordinal scoring systems.

Among the studies that evaluated the effects that maceration on subsequent forensic examinations, the majority (six in total, [36, 37, 39, 40, 42, 43]) focused on DNA quality and preservation, with about half also comparing the efficacy of different maceration methods more generally. Two studies assessed the impact of maceration on bone metabolomics (proteome and lipidome) [44, 45], one focused on bone fluorescence [46], one on bone microstructure and hardness [41], and one on stable isotope analysis [47]. Only four studies assessed or commented on the effects of maceration on sharp force trauma analysis [31, 32, 35, 37].

Two studies evaluated the effects of maceration on DNA quality [43] and 3D geometry [48] but focused only on a single maceration method, i.e. water bath maceration without additives, without further exploring the possible effects of other methods on these aspects.

The studies presenting novel maceration methods for use in forensic setting (without any comparative testing; seven in total) were published between 1975 and 2021. Most of the older studies were more inclined towards the use of more aggressive methods such as bleach solutions or chemical mixtures. In more recent studies, microwaves have been proposed as an alternative maceration method [31]. The method received mixed opinions regarding quality and ease of use in subsequently published articles. These include concerns about size limitations, overheating issues, and instances of tissue remaining more adherent than expected during maceration cycles. Other approaches described in literature are the use of an oven preheated to 100 °C, followed by a combination of water bath, bleach and detergent [32], as well as the use of different instruments such as a steam kettle [33] or incubator [34].

Published studies reported varying maceration times for the same maceration method, with temperature varying significantly between studies for the same method. The microwave technique was judged as one of the quickest methods. Maceration with sodium hypochlorite was judged by most of the studies as being very quick, taking even 15 min to finalize the removal of soft tissue. Despite its efficiency in terms of time, sodium hypochlorite has shown adverse effects on bone quality, health and subsequent analyses. Similarly, maceration with borax has been considered too aggressive, affecting bone preservation and quality. Most studies agreed that maceration with detergents or the use of heated water bath (at approximately 80 °C) are preferred for forensic use, the latter being particularly favored as it is free from additives that can impact different bone aspects. However, Steadman et al. [36] have commented how prolonged soaking could be potentially harmful to bone DNA preservation, preferring thus brief, high-intensity methods, such as sub-boiling and boiling. Studies have also shown that even maceration methods that were previously deemed “safe and gentle” can impact the bone proteome [44, 45], which may influence the outcomes of any subsequent analyses conducted on the bones. Maceration methods have also been found to alter stable isotope ratios, potentially leading to erroneous geolocation of remains if analyses are performed after maceration [47].

In the reviewed publications, various types of equipment have been used for maintaining a consistent temperature during the maceration process, including slow cookers, ultrasound baths, sous-vide devices, steam kettles, incubators and hotplates (including the use of magnetic hotplate stirrer).

A summary of the conclusions drawn from all reviewed studies is presented in Table 3.

**Table 3:** Summary of commonly tested maceration techniques, outlining their main pros and cons

Method	Pros	Cons
Bleach	Fast (nb: some studies reported no effect on dissolution of soft tissue) Easy removal of soft tissue Effective when carefully controlled	Can cause decalcification and powder formation Extensive surface damage and increase in porosity described Caustic nature continues even after processing May produce artifacts Poses health and safety risks Affects DNA preservation Affects the retention of cut marks
Detergent in heated water bath	Relatively fast and safe Economical Good DNA preservation reported in studies No effects on bone appearance Has been judged most successful for maceration of neonatal remains, together with temperature-controlled heated plain water bath (nb: in textbooks cold water maceration is suggested for processing infant and fetal remains)	Incomplete ligament dissolution (dish soap maceration) Some effects on bone quality reported when high temperatures (90 °C) were used Affects the metabolome preservation (nb: metabolome preservation was investigated just in maceration with detergents) May include additives that can affect the bone
Enzymatic	Effective and fast Gentle on surface morphology The use avoids the presence of additives	Relatively expensive in comparison to other methods Requires technical knowledge as being deemed “unpredictable and invasive” (these risks are deemed minimal if enzymes are used as solution) Discoloration observed Bone weakening reported Affects DNA preservation Varying results in retention of cut marks reported
Plain water—room temperature	Preserved bone texture Simple nb: heated water has been judged to be best for maceration of burnt remains	Very slow Strong odor caused by bacterial activity Affects DNA preservation
Plain water- boiling	Ease significantly tissue removal Good DNA preservation (due to short exposure times)	Affects bone edges (fraying), compromise cortical surface (nb: even subboiling temperatures can negatively affect the bone integrity) Risk of heat induced microcracks Affects the stable isotope values (nb: all methods involving heated water (90–100 °C) affected it) Varying results in retention of cut marks reported

### Survey

#### Legal medicine, forensic anthropological institutes, archaeological and anatomical institutes (FM)

**Fig. 1:**
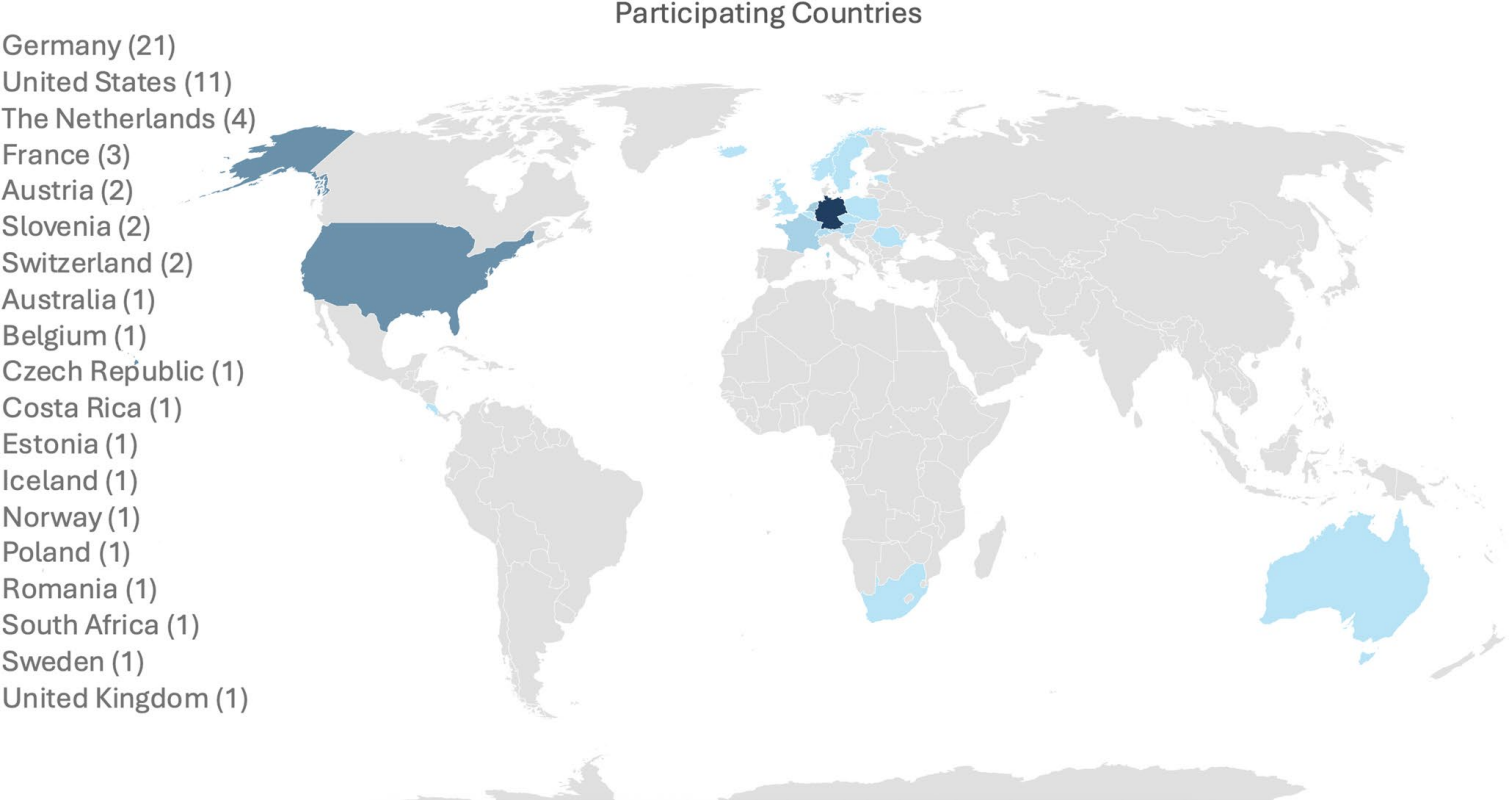
Countries that participated in the survey; FSL and FM included

Overall, 54 institutes worldwide registered in the survey. Most of them are located in Germany (24) and the USA (12). Other participating institutes are from France (4), South Africa (2), Australia (1), Austria (1), Belgium (1), Costa Rica (1), Denmark (1), Iceland (1), Romania (1), Slovenia (1), Sweden (1), Switzerland (1), The Netherlands (1) and the United Kingdom (1). Of the 54 participants, 18 left the questionnaire unanswered after registration, leaving 36 evaluable answers for the analysis (Germany (11), USA (10), France (3), South Africa (1), Australia (1), Austria (1), Belgium (1), Costa Rica (1), Iceland (1), Romania (1), Slovenia (1), Sweden (1), Switzerland (1), Netherlands (1), United Kingdom (1)) (Fig. 1).

Of all the institutes 27 are working exclusively with human remains, whereas 5 working with both human and non-human remains. Four of the institutes did not answer this question. 

**Fig. 2:**
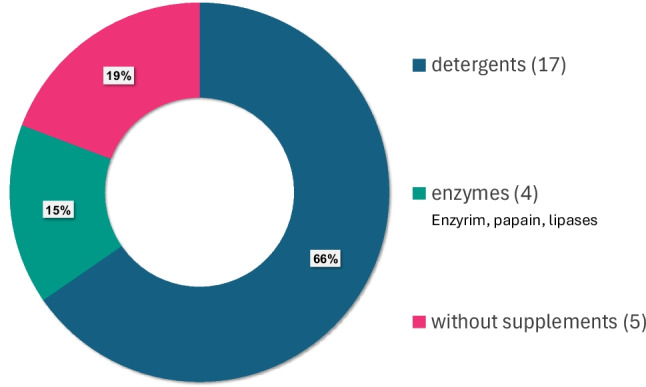
Maceration methods reported by surveyed institutes worldwide

*Maceration methods*: All of the institutes conduct maceration by soaking the bone in water baths with or without the use of additives (Fig. 2), with nearly half (17) using detergents as supplements to the water bath.

*Temperatures*: The indicated temperature intervals for the maceration procedures span from room temperature to 100 °C (boiling point), but most institutes opt for a temperature between 60 and 75 °C (14).

*Post-maceration treatment for bones*: Nine institutes incorporate a separate degreasing step in their maceration process. The degreasing is most commonly performed using acetone or isohexane. Two institutes are using an extra degreaser (special degreasing equipment; degreasing solvent is vaporized to remove grease).

Almost one third (11) of the institutes whiten the bones after maceration, all of them using hydrogen peroxide (a milder oxidizer than sodium hypochlorite) for this purpose. Sodium hypochlorite was reported as an alternative bleaching agent by one institute.

*Equipment*: most institutes use a stainless-steel basin (20). Only one institute is currently working with a polymer bucket. An incubator is used by six institutes to maintain the temperature constant. Additional equipment used includes simple heating pots (3), slow cookers (3) and a steam kettle (1).

**Fig. 3:**
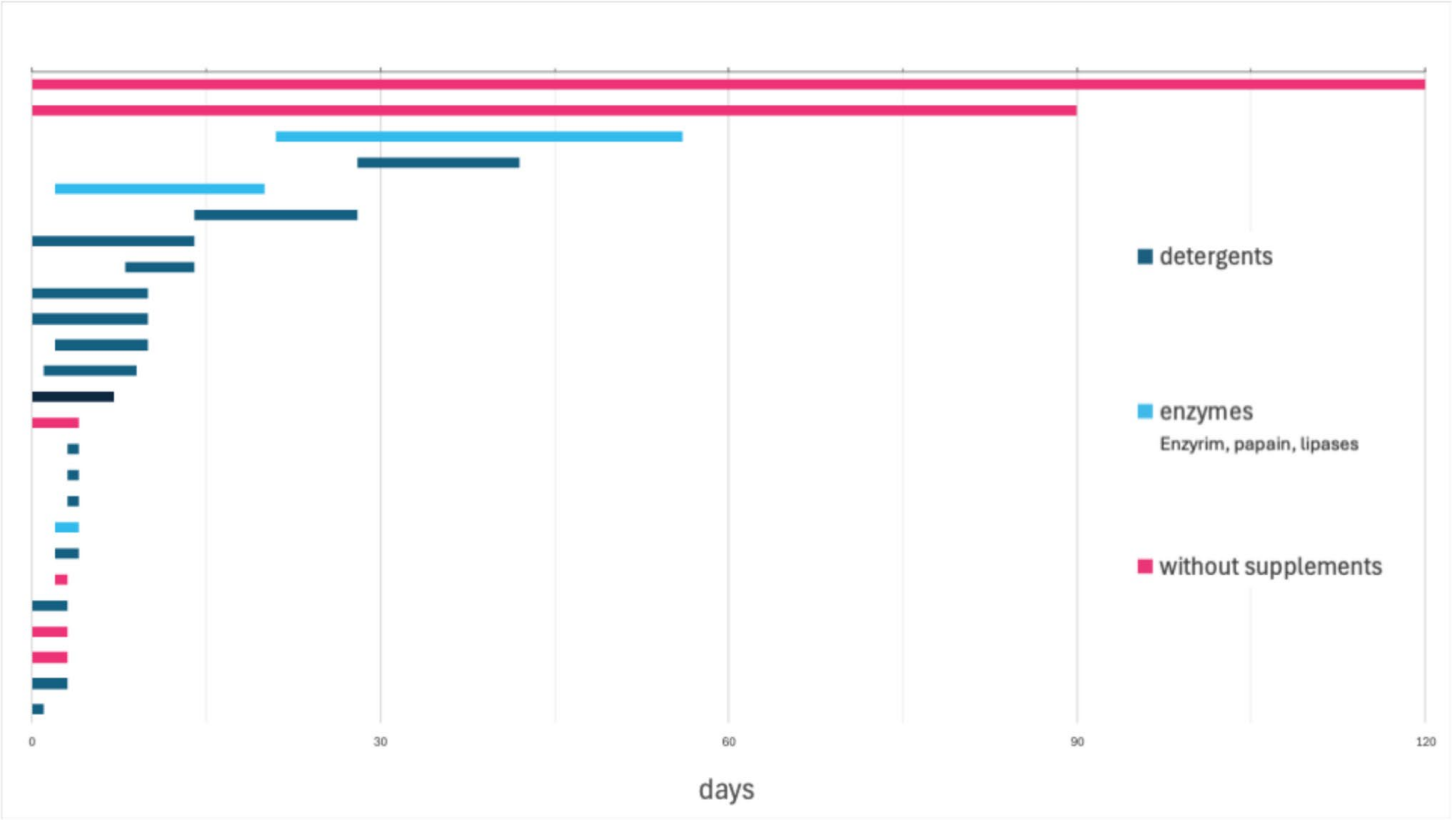
Duration of maceration processes for macerations that take min. 24 h; grey: n.a. (1)

*Duration of maceration*: reported maceration times varied considerably (Fig. 3). The fastest macerations have been reported to be completed in as little as 15 min (methods relying on the use of enzymatic detergent), whereas the more time-consuming method has been reported to take up to four months (the use of cold-water maceration without additives). Five institutes reported maceration times of less than one day (not included in Fig. 3 for clarity). Those institutes made use of Papain, (boiling water with) heavy duty detergent, or enzymatic detergent to macerate their remains.

**Fig. 4:**
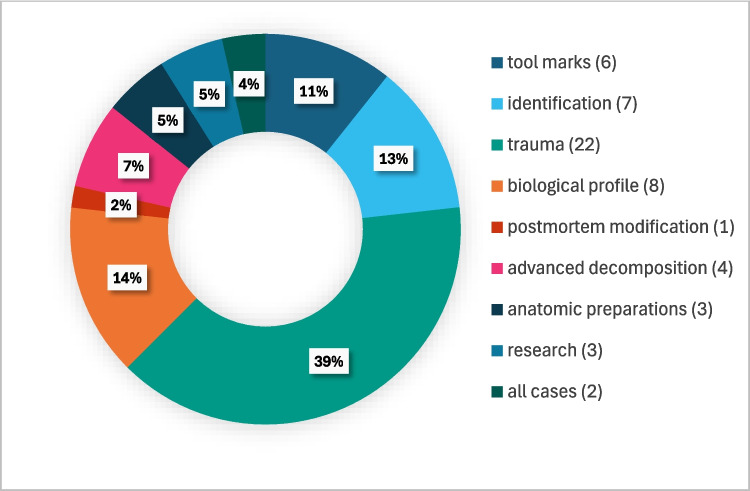
Maceration purposes reported by surveyed institutes worldwide

*Purpose of maceration*: Most of the institutes (22) perform macerations when trauma to the bone should be analyzed, while other focus on other aspects of the forensic anthropological analysis (Fig. 4). The four anatomic institutes macerate remains for anatomical preparations and research purposes.

*SOP*: only half of the questioned institutes (18) have a Standard Operating Procedure (SOP) in place related to maceration, 11 institutes agreed on making these SOPs available for research purposes. So far only four institutes sent their SOPs.

Out of the 18 SOPs, 13 take the preservation of tool marks into account, while only nine address the preservation of adhering material traces. Three of the reviewed SOPs take neither tool marks nor material traces into account during maceration.

#### Forensic Science Laboratories (FSL)

In the survey addressed to forensic labs, 21 laboratories from Europe (20) and the USA (1) took part.

Out of the 21 laboratories, only three carry out macerations themselves. Of these, two retain an SOP for the maceration that addresses tool marks as well as material traces.

*Maceration methods*: Since one of the three laboratories that carry out macerations have not shared information of the used method, only two methods are presented herein. One laboratory uses a water bath at 75 °C combined with a heavy-duty detergent. The maceration process takes several hours. The other laboratory macerates human remains and uses different protocols depending on whether the samples are classified as large or small. Larger specimens are macerated at 80 °C, whereas smaller ones are macerated at 60 °C, both in a water bath with enzymes (lipase and papain), and afterwards degreased with Supralan (a washing detergent and degreaser). Maceration of smaller specimens takes one day, whereas larger specimens need several days for full removal of soft tissue.

**Fig. 5:**
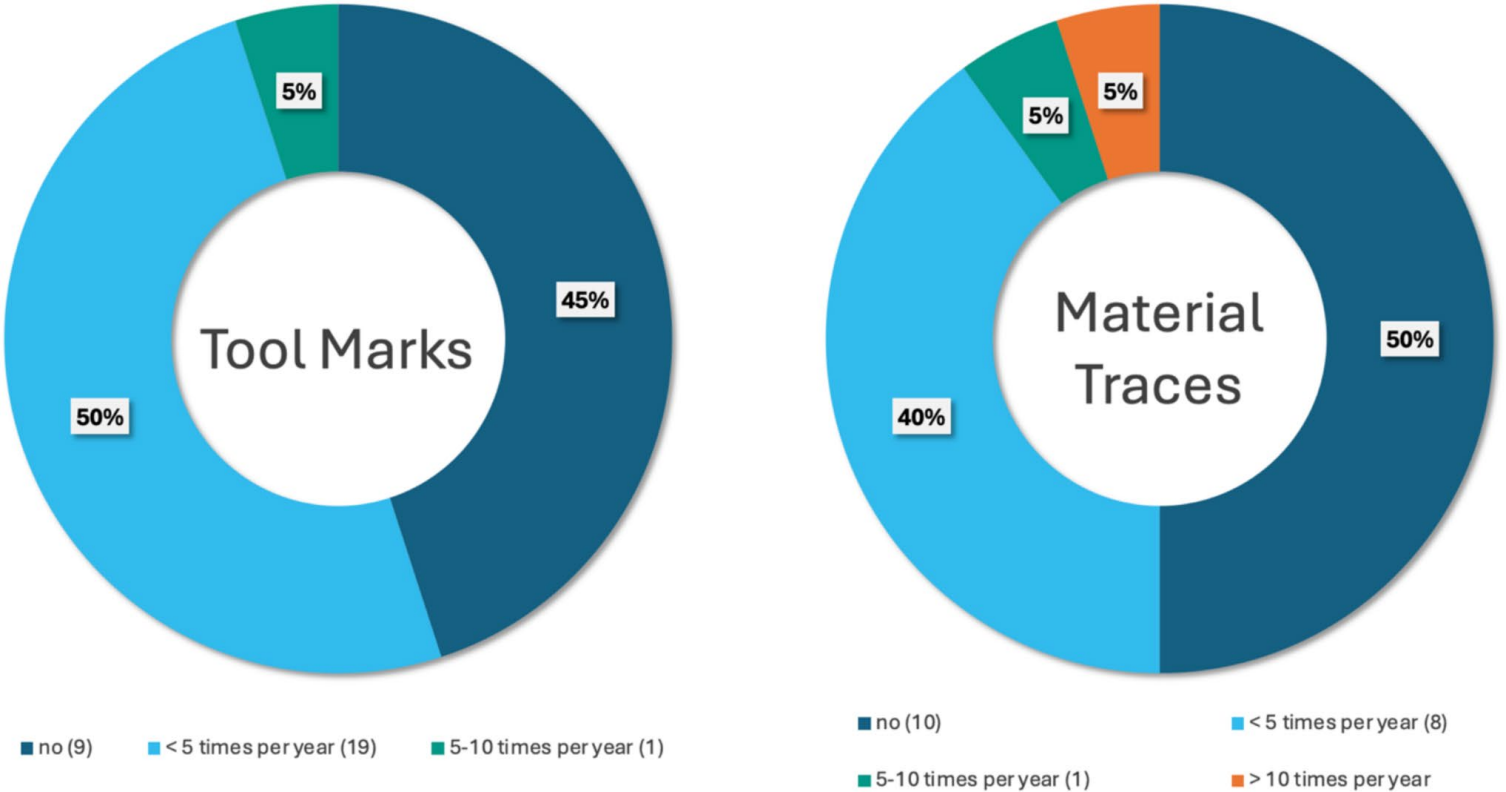
Reported number of Examinations of Tool Marks (left) and Material Traces (right) on Human Bone and Cartilage per year

*Frequency of tool mark and trace analyses*: Tool mark examinations on human bone and/or cartilage are performed by 11 out of the 21 laboratories with varying frequency (Fig. 5 left). Likewise, 11 laboratories reported investigating material traces on human bone and/or cartilage, with most performing fewer than five such analyses per year (Fig. 5 right).

*Methods used for securing evidence before maceration*: to secure tool marks before macerations the following methods have been reported: photography (8), casting (7), 3D scans (2). When material traces are examined directly on the bone or cartilage (in situ, prior to maceration), the following methods and tools are used: tweezers (3), evidence film (2), wooden sticks (1) and casting material (2).

*Methods used for documenting/sampling evidence after maceration*: After maceration the tool marks are documented by casting (9), photography (6), 3D scans (4) and digital microscopy (1). After maceration, material traces are collected using tweezers (4), evidence film (1), wooden sticks (1) and scraping (1). For both (before and after maceration), a combination of more than one tool is used by the laboratories.

*Alterations reported after maceration*: three out of the 21 laboratories reported changes in tool marks due to maceration. Observations included increased bone fragility, changes to trace edges, and indistinct or lost mark when using washing detergents for maceration. Two (out of ten) laboratories reported trace alterations due to maceration. These included false traces, trace loss or changes in existing traces. One laboratory described significant surface changes, which in severe cases led to breakouts of the compact bone, while the second observed the loss of trace particles when using enzymatic maceration.

## Discussion

Maceration has been a topic of research and methodological development in forensic literature over the years. It has gained more steady attention in the last two decades, likely driven by the increased awareness of the need for validity and reliability of methods and laboratory procedures used, and for the accreditation of forensic laboratories [51]. The literature published to date [24–50] reflects the efforts to identify those maceration methods that would best meet the key requirements for forensic application, such as the preservation of bone integrity, cost-effectiveness, accessibility to materials, ease of application and minimal risk of introducing artifacts compromising subsequent analyses or posing health risks to practitioners [28, 31, 38].

The analysis showed that different methods have been compared through years, with enzymatic-based methods [27, 29, 31, 35–37, 41, 42, 46] detergent-based methods [24, 28, 32, 34–40, 42, 44–47, 49, 50] and plain water methods [31, 33–36, 39–43, 46–49] emerging as the most commonly tested. The latter two are most often preferred due to their ease of application and the availability of the substances involved [31, 50]. A detailed comparison of study results remains challenging, as even when the same maceration method was employed, the studies differed in the material processed (human-non human; different bone types), equipment used (microwaves, slow cookers, ultrasound baths, sous-vide devices, steam kettles, incubators and hotplates), temperatures applied (15–100 °C), and substances employed (e.g., different enzymes, various brands of detergents, each with distinct composition, or differing dilutions of chemicals). Certain studies employed predefined time frames for maceration, regardless of the outcome. Consequently, the reported times and results varied, which may have led to varying interpretations of the same method across studies.

The literature review has highlighted that no maceration method is without drawbacks [24, 31, 50]. Even the methods deemed to be effective and safe have shown to cause issues for specific subsequent analyses (such as DNA or proteome) [43–45, 47], particularly if applied for prolonged periods of time. Similarly, prolonged exposure to heat has an adverse effect on bone quality. A growing interest in evaluating the impact of maceration on aspects of bone that have previously not been thoroughly examined has emerged in recent years, showing an awareness that bones can become an important source of information in forensic investigations, particularly with the development of new methods and analyses. Studies published to date have primarily focused on the effect of maceration on DNA preservation [36, 37, 39, 40, 42, 43] and more recently the metabolome [44, 45]. Other areas of potential importance for the forensic investigation remain however under-researched or just peripherally addressed (i.e., the impact of maceration on tool mark analysis and material trace analysis).

The survey, conducted to clarify which maceration techniques are actually used in practice, revealed both similarities and differences in their application across forensic laboratories and institutes.

The survey results are largely representative of practices in Europe, with 67% FM and 95% FSL located in Europe, and nearly half of the respondents based in Germany.

The results show that maceration is primarily conducted by forensic medicine institutes (FM), which likely process the remains before sending the bones to forensic laboratories for subsequent analyses (tool mark analysis, material trace analysis). Despite most FSL group respondents being tool mark experts from Europe (17), nearly half of the surveyed laboratories still do not examine tool marks or material traces on human bone and cartilage. This suggests that maceration is primarily performed for the exclusive use of forensic departments/institutes, which reported performing tool mark analyses by themselves (16%), beside routine trauma examination.

The survey results indicate that the methods performed in practice align quite well with those presented in published studies, showing consistency between applied techniques and existing research.

Most of the institutes use methods considered acceptable by the currently published literature, with detergent-based maceration being the most commonly applied procedure. Detergents combined with hot water are known to speed up maceration and, after plain water maceration, appear to have the least potential for causing bone alteration [24, 28, 35, 39]. Detergent, most of which contain lipases, facilitate degreasing of bone, eliminating the need for separate degreasing steps. Additionally, detergents are safer for both health and the environment compared to harsh chemicals such as borax or bleach [24, 26, 31] and they are also more cost-effective compared to enzymatic solutions. The reported detergents however vary widely and span from heavy duty detergents, laundry detergents to dish soaps, suggesting differences in their composition. The extent to which practitioners consider the chemical composition of these detergents, which can also affect negatively the skeletal material [29, 37, 52], remains unclear from the survey. For instance, some heavy-duty detergents contain sodium perborate, which breaks down in water into hydrogen peroxide and sodium bicarbonate, potentially causing a bleaching effect and eventual damage on bones.

The temperatures reported in the survey align with those preferred in the literature, with most institutes using temperatures between 40–60 °C, which is considered optimal for enzymatic activity [50, 53].

The water bath maceration without additives, which some studies and institute deem to the method least likely to alter bone [34, 54], has also shown to be highly time-consuming [34, 35], sometimes taking several months before completion. Interestingly, some institutes claim to perform water bath macerations without additives in under a week. This and other variations in duration of the maceration process can be explained not only by the method itself but also by factor such as sample size, degree of soft tissue present and decomposition.

Interestingly, despite the well-documented adverse effects of boiling water [31] and aggressive bleaching methods on bone integrity [24, 31], some institutes continue to use water baths at 100 °C.

Several institutes are whitening their bones even for forensic purposes, often to preserve the specimen showing specific lesions, which are crucial for reconstruction or presentation in court and may not require further subspecialized examination. However, it should be kept in mind that in all cases of bone trauma, additional analyses may still be requested at later stage. Heavy bleaching can alter the bone and therefore affect the tool marks appearance and features. Bleach may also react with material traces adhering to the bones surface, which could lead to their loss of creation of false traces. On the other hand, both boiling and the use of bleach can be used to help eliminate biohazard risks or inhibit the growth of bacteria and mold [55].

Significant differences in maceration techniques between European countries and the USA have not emerged. Comparison between other countries was not possible, as there were not enough survey responses to conduct such analysis.

None of the surveyed institutes reported applying the novel maceration methods described in the literature, such as microwave maceration [31, 36, 38, 42]. Whether this is due to a lack of verification and standardization of the method in literature, or simply limited awareness of these techniques, remains unclear, underscoring the need for further testing to evaluate their suitability for routine forensic use. Other recently described methods should also be tested in future studies [32–34].

An important and concerning finding of this study is that many surveyed institutes lack an SOP for maceration, meaning that no standardized maceration procedure is incorporated in most laboratories. The absence of standardization may lead to inconsistencies in practice, reduced comparability of results, potential loss or alteration of evidence, and challenged to meet forensic quality standards.

In summary, this study highlights the current practices and challenges associated with maceration methods in forensic contexts, revealing both alignment with published literature and important areas for improvement. While most institutes use methods deemed acceptable in the literature, such as detergent-based maceration, the yet undetermined effects of chemicals on bone microstructure, non-harmonized settings with lack of process control and absence of standardized operating procedures (SOPs) highlight the need for more research and broader discussion to make the use of maceration safer and more reliable.

The literature acknowledges that maceration techniques have their own advantages and disadvantages, which must be carefully assessed before being applied in a forensic case [29, 31, 50]. No single maceration method is universally preferred, as the chosen approach often depends on factors such as the type of bone, the condition of the remains, case-specific characteristics, and the forensic questions to be addressed, as noted by King et al. [31]. Regardless of these variations, certain principles should always be upheld, and clear guidelines, while allowing for case-specific flexibility, should be in place [56].

Today, some efforts in making guidelines e to serve as a reference have been made. Already in 2011, the Scientific Working Group for Forensic Anthropology (SWGANTH) has summarized these principles in its recommendations [57]. At present, there are still no formally published guidelines on skeletal preparation. The *Organization of Scientific Area Committees for Forensic Science (OSAC)* has drafted a proposed standard, which is currently available online [22]. This standard, after review by the Academy Standard Board (ASB) Anthropology Consensus Body, is expected to be made available for public comment soon (as ASB Standard 225) [58].

In practice, priority should always be given to methods proven to be the most effective while being the least harmful to bone structure, form, dimensions, and mechanical properties. This remains important even when such precautions might seem unnecessary for the immediate purpose of maceration, as additional sampling or unplanned analyses may be required at a later stage. The choice of method should be informed by findings on the efficacy, validity and limitations of each technique as presented in literature. Unfortunately, this knowledge remains insufficient in certain areas, especially for the impact that various maceration techniques can have on tool marks and material traces on human bone.

Further research into maceration and its effects on different forensic analyses is thus essential, as it is also evident that a standardization in form of an SOP across laboratories is needed. Together, these efforts will ultimately ensure that appropriate methods are implemented, that processes and results can be reliably replicated, effective techniques shared and adapted between laboratories, and maceration methods can be systematically compared and improved.

## Appendix A

**Table 4:** Literature review findings summary

	**Author**	**Additional aims**	**Material**	**Method**	**Temperature**	**Conclusions/Comments**
Presentation of maceration methods	Snyder et al., [25]	-	Human remains	**Antiformin** solution (sodium carbonate and bleaching powder)	close to boiling	Later studies have highlighted health risks, the risks associated with prolonged heating, and the risk for bone dissolution
	Stephens, [26]	-	Skulls (human)	**Sodium hypochlorite** diluted in water (up to 10%) (cycles)	50–70 °C	High concentrations of sodium hypochlorite can lead to decalcification and powder formation
	Grundmann and Röetzscher, [27]	-	Jaws (human)	Enzymes (***ENZYRIM***) in combination with concentrated detergent in an ultrasonic water bath	60 °C	The method has been deemed as cost-effective (tissue removal was achieved after 2 h)
	Fenton et al., [28]	-	Human remains	Water bath with **powdered detergent** (*Alconox*) and **powdered sodium carbonate**	simmering	The methods were deemed fast, safe and economical and produced high quality, degreased skeletal elements
	Simonsen et al., [29]	-	Whole mice/rats	Water bath with **commercial enzymes** (protease, lipase) in a **magnetic hotplate stirrer**	55 ± 5 °C	The *enzymatic* method was deemed fastest The use of a hotplate stirrer was deemed to significantly reduce maceration time and eliminate the effects of additives present in detergents The method left a reddish color on bone
	Mann and Berryman, [30]	-	Rib cage (human)	**Undiluted household bleach** (sodium hypochlorite) and afterwords immersion in tap water	RT	The method was considered safe, effective, and efficient when DNA extraction was not required
	King et al., [31]	Testing, comparison, and evaluation of maceration methods Impact of maceration on sharp force trauma analysis	Ribs (porcine)	**Microwave**	-	The *microwave* method was judged to be successful in terms of time, bone and detail preservation No maceration technique fully satisfied all the analyzed criteria *Sodium hypochlorite* caused extensive surface damage but offered the quickest and easiest soft tissue removal
				Water	75 °C, 100 °C	
				Enzymes (pancreatin)	35 °C	
				Sodium hypochlorite (4,9%)	68 °C	
	Husch et al., [32]	-	Ribs and hindlimb- femur (porcine)	Combining **baking in oven** (100 °C) wrapped in aluminum foil with/without dishwasher (*W5*) and **water maceration with bleach and detergent**	80 °C	The method has been judged time-saving and cost-effective, degrading soft tissue after 5 h of baking and 1 h of water bath maceration
	Armelli et al., [33]	-	Human remains	**Steam Kettle** skeletal preparation	90 °C	The method has been deemed efficient requiring minimal disarticulation, no chemical agents, and minimal intervention or attention by the processor
Testing, comparison and evaluation of maceration methods	Mairs et al., [35]	Impact of maceration on Environmental Scanning Electron Microscopy (ESEM) analysis	Forelimbs (porcine)	Water	RT, 40 °C, 60 °C	*Detergents at medium temperatures* resulted in best cleaning results (Persil, Daz, and Ariel took 2 days at 60 °C to remove tissue) *Plain water maceration* showed no significant effect on soft tissue removal after 4 days No loss of detail was observed under ESEM
				Water bath with detergent in tablet form (*Persil, Bold, Daz, Vanish, Sunfresh, Ariel, Fairy*)	RT, 40 °C, 60 °C	
				Water bath with enzyme (protease- papain)	RT, 40 °C, 60 °C	
	Steadman et al.,[36]	Impact of maceration on DNA	Ribs (porcine)	Microwave	-	*Detergent methods*, *hot water methods*, and maceration by *boiling water* proved to be the most efficient in terms of time, ease of processing, and bone quality, yielding the highest amount of total DNA Maceration at r*oom temperature*, *hydrogen peroxide*, *bleach*, and *EDTA/papain* maceration resulted in low DNA recovery
				Water	RT, 90 °C, 100 °C	
				Water bath with detergent (*Biz*)/sodium carbonate	< 90 °C	
				Enzyme (Papain) and EDTA	< 45 °C	
				Water bath with detergent (*Biz*)/sodium carbonate	< 90 °C	
				Sodium hypochlorite (10%)	RT	
				Water bath with hydrogen peroxide (3%)	RT	
				Water bath with meat tenderizer/soap (*Palmolive*)	< 90 °C	
	Uhre et al., [37]	Impact of maceration on DNA Impact of maceration on sharp force trauma	Ribs (human)	Water bath with washing powder (*Ariel*)	100 °C	*Enzymatic methods* were quickest, completing tissue removal in approximately 3 h *Boiling* resulted in frayed bone edges Bone texture and DNA were preserved with both methods
				Water bath with enzymes (lipase, protease)	55 ± 5 °C	
	Couse & Connor, [38]	-	Skull (puma), Paws (bear), Human remains	Microwave	-	*Water bath with cleaner/degreaser* scored the best The *dish soap method* had issues with softening ligaments The *microwave method* was considered the least useful and most time-consuming
				Water bath with dish soap (*Dawn*)	100 °C	
				Water bath with cleaner/degreaser (*Greased Lightning*)	100 °C	
	Mahon et al., [50]	-	Hindlimbs (pigs)	Water bath with detergents (Surf, Ariel, OMO, Sunlight, Sunlight dishwashing liquid)	45 °C, 50 °C, 55 °C, 60 °C	*Detergents* showed no direct effect on bone appearance chalkiness resulted from the temperatures applied The optimal temperature was identified as 60 °C *OMO Auto* was the most suitable agent due to the absence of aggressive ingredients such as bleaching agents
Testing, comparison and evaluation of maceration methods for specific purposes	Triaca et al., [49]	Maceration for burnt remains	Trotter (porcine)	Water	35 °C, 80 °C	*Plain tap water* maceration at *80°C*for 24 h was judged to be the best method, as it showed no damage to the bone and resulted in the least amount of fragmentation
				Water bath with sodium hypochlorite	15 °C ± 2 °C	
				Water bath with dishwashing agent (*Sunlight 100*)	15 °C ± 2 °C	
				Water bath with powder detergent (*Omo*)	15 °C ± 2 °C	
				Water bath with dish soap (*Dawn*)	100 °C	
				Water bath with cleaner/degreaser (*Greased Lightning*)	100 °C	
	Keyes et al. [24]	Maceration for neonatal remains	Neonate pigs	Water bath with laundry detergent/sodium carbonate	75 °C	*Laundry detergent* maceration took the longest but was deemed most successful B*leach* caused no significant changes to soft tissue *Sodium hypochlorite* and *borax* solution caused artifacts and emitted dangerous fumes
				Generic household bleach	RT	
				Sodium hypochlorite	RT	
				Borax solution	75 °C	
	Dunn et al., [34]	Testing, comparison and evaluation of maceration methods for specific purposes—maceration for neonatal remains	Human remains (neonatal)	Water	RT	*Incubation* was deemed the best method for forensic maceration, taking a few days for maceration but preserving bone quality *Room temperature* water (cold-water) took the longest time for maceration (4–5 weeks) but preserved bone texture *Hot water detergent maceration* required the least time but negatively impacted bone quality *Dehydration* preserved cartilage; however, the removal of soft tissue was difficult. A combination of cold-water maceration and dehydration was deemed a better solution when time allows
				Water bath with powered detergent and sodium carbonate	90 °C	
				**Dehydration** (food dehydrator)	60 °C	
				Water bath- **incubator**	78 °C	
	Arismendi et al., [39]	Impact on the analysis of DNA	Ribs (human)	Water	simmering	The chemicals did not appear to affect the subsequent DNA analysis Exposure to excessive heat seem to adversely affects the DNA analysis Simmering water combined with Alconox was found to be the most effective method for processing bone samples to obtain satisfactory results
				Water bath with detergent (*Alconox*)	simmering	
				Water bath with washing soda (Arm&Hammer®)	simmering	
				Water with later add of borax	intermittent boiling (100 °C) and cooling	
				Water bath with detergent (*Alconox*) and later concentrated degreaser	simmering	
Impact of maceration on the bone analysis and features	Rennick et al., [40]	Impact on the quality and quantity of DNA	Human, non-human bones	Boling water	100 °C	Bleach caused lower yields, while the detergent/carbonate method allowed the largest segments of DNA to be amplified
				Bleach		
				Water bath with powdered detergent/sodium carbonate		
	Yin et al., [41]	Impact on bone microstructure and microhardness	Hindlimb-femur (lamb)	Water bath with enzyme (protease—trypsin)	RT	No differences in porosity were observed between maceration methods, but enzymatic maceration showed evidence of bone weakening
				Water	RT	
	Lee et al., [42]	Impact on the quality and quantity of DNA	Lower limbs (human)	Microwave	-	The *microwave* method was the quickest (up to 38 min), followed by the *detergent/sodium carbonate mixture* (6 h) The *enzymatic* maceration and *bleach* required several days, while *hydrogen peroxide* took the longest (> 100 days) *Microwaving* and the *detergent/sodium carbonate mixture* caused the least damage to DNA recovery and amplification
				Water	< 90 °C, 100 °C	
				Water bath with detergent (*Biz*)/sodium carbonate	< 90 °C	
				Enzyme (Papain)/EDTA	45 °C	
				Sodium hypochlorite (10%)	RT	
				Water bath with hydrogen peroxide (3%)	RT	
				Water bath with meat tenderizer/soap (*Palmolive*)	< 90 °C	
	Frank et al., [43]	Impact on DNA recovery	Human bones	Water	62 °C	*Hot water maceration* for 45 min caused some genetic loss, though not statistically significant compared to the control The method posed some challenges in producing a complete DNA profile
	Bonicelli et al., [44]	Impact on the bone proteome	Hindlimb-tibia (bovine)	Deionized water bath with dish soap	55 °C	*Both methods* resulted in a significant reduction in metabolites and lipids, accompanied by contamination
				Deionized water bath with laundry detergent	87 °C	
	Gent et al., [45]	Impact on the bone proteome	Hindlimb- tibia (bovine)	Water bath with dish detergent	55 °C	*Both methods* affected the integrity of the bone proteome, with higher temperature methods having the greatest impact Lower temperatures and shorter submersion times, without the use of detergents, should be preferred
				Water bath with laundry detergent	87 °C	
	Maidment et al., [46]	Impact on bone fluorescence	Ribs (porcine)	Water	80 °C	*Hot water maceration* preserved collagen best and provided consistent fluorescence W*ashing powder* was deemed to be the most destructive *All methods* increased fluorescence *Enzymatic maceration* was quicker (1 h) than hot water maceration (3.5 h)
				Water bath with washing powder (*Surf*)	50 °C	
				Water bath with enzymes (protease, lipase)	55 °C	
	Ceja et al., [47]	Impact on Stable Isotope values	Ribs (pig)	Sodium hypochlorite (10%)	RT	Maceration methods involving heated water have significantly altered the ratios of the stable isotopes, and can thus contribute to erroneous geolocation of remains
				Water	100 °C	
				Water	90 °C	
				Water bath with dish soap and meat tenderizer	90 °C	
				Water bath with detergent (Alconox) and sodium carbonate	90 °C	
				Water bath with degreasing detergent	90 °C	
	Bittner-Frank et al., [48]	Impact on 3D geometry of bone	Forelimb (human)	Water	60 °C	After maceration in water for two weeks, the bone showed a shrinkage of 0.12 mm

Some of the authors also tested invertebrate skeletal preparation methods, as well as mechanical and power washing methods, to remove soft tissue from bones. These methods and their results are not included or discussed in the table, as they were outside the scope of this study that focused on maceration in the narrow sense (“the removal of soft tissue from skeletal remains through prolonged immersion in a liquid bath“). Post maceration procedures (e.g. additional degreasing) have also not been included in the table.

When authors did not specify the temperature and simply used the term 'simmering' (some included the temperature, approximately 90 °C), the term 'simmering' was recorded in the table.

RT – room temperature.
